# Influence of inhibition on encoding vocalizations in the mouse auditory midbrain

**DOI:** 10.1186/1471-2202-13-S1-P72

**Published:** 2012-07-16

**Authors:** Alexander G Dimitrov, Graham I Cummins, Zachary M Mayko, Christine V Portfors

**Affiliations:** 1 Department of Mathematics, Washington State University Vancouver, Vancouver WA 98686, USA; 2 School of Biological Sciences, Washington State University Vancouver, Vancouver WA 98686, USA

## 

Inhibition is well known to shape responses to sensory stimuli. In the auditory system, it can affect frequency response curves and responses to complex stimuli. Here, we examined how inhibition affects response to vocalizations inferior colliculus (IC) of female CBA/CaJ mice. We studied two cases in awake mice: normal auditory processing (control) and auditory processing after pharmacological blocks of inhibition (test, application of bicuculline and strychnine to block GABA A and glycine receptors). We observed several types of response across 23 tested cells. Four cells were not stimulus responsive, but their spontaneous firing rate did increase in the test condition. Three cells generated distinct stimulus-dependent responses that did not change significantly in the test condition. One cell changed its response pattern significantly in the test condition. Six cells responded only to one or two stimuli. These cells maintained the same selectivity, but on average increased their firing rates in the test condition. The remaining cells followed a pattern where responses present in the control condition also occurred in the test condition, with similar temporal pattern, but with more, or more reliable, spikes. In many of these cells additional responses occurred in the test condition in response to stimuli that generated no response in the control condition. This is consistent with the hypothesis of a single response structure in both conditions, more of which exceeds threshold in the absence of inhibition, but in the case of changing from no visible response to some response, we cannot rule out a change in the underlying code. We do establish that the degree of temporal precision required to discriminate different response patterns is typically the same in both conditions, and that the responses to groups of stimuli show similar structural relationships in both conditions, within the subset of stimulus conditions that generate some response in both conditions. Our observations are thus mostly consistent with inhibition changing the overall excitability of cells, but not changing the underlying stimulus-dependent response patterns.

We evaluated cells using inspection of the response rasters, spike-counting measures, stimulus/response mutual information, and hierarchical clustering based on several event sequence distance measures. We measured the rate of mutual information loss with increasing amounts of noise in the timing of response rates. We take this rate of loss to indicate the degree to which the stimulus encoding depends on the precise timing of response events. We used clustering of the stimuli, based on their sets of evoked responses, to evaluate the similarities between the structures of response to various vocalizations. We measured the rate of loss of mutual information with increasing amounts of noise in the timing of response rates. This rate of loss indicates the degree to which the stimulus encoding depends on the precise timing of response events.

